# New Appliances and Things Medical

**Published:** 1904-08-06

**Authors:** 


### NEW APPLIANCES AND THINGS MEDICAL.

### CLARKE'S NURSERY MILK.

(J. Clarke and Sons, 46 Hill Rise, Richmond.)

This seems to be a milk of very good quality. It is obtained from carefully selected Jersey cows. It is rich in fat and well up to the standard in all respects. It is supplied in bottles containing half a pint, which are closed by air-tight stoppers. The taste of the milk is good, and when treated with acid and pepsin it forms a friable coagulum, from which fact it may be assumed that it is easily digestible. Unopened, the milk keeps well, but soon goes sour subsequently. We think the milk is distinctly to be recommended.

### GALAK.

(The Galak Milk Products Co., 118 Fenchurch Street, E.C.)

We have received two samples of this substance. One in a powder form, contained in tins, the other in the form of a tabloid. Both these are essentially the same, and consist of the substance to which the name galak has been applied. Galak is a cream-coloured granulated powder consisting exclusively of the solid constituents of sweet milk. This powder, mixed in the proportion of two tablespoonfuls to half a pint of hot water, makes an opaque liquid resembling in appearance ordinary milk. This milk is produced by the Just-Hatmaker process. The advantages claimed for the product are its form, rendering it exceedingly easy of transit, its freedom from all bacteria, its uniformity of composition, and, lastly, that even when opened it will keep for an indefinite period. When added to water in the proportion indicated the resulting fluid has approximately the composition of milk. Physiological experiments have been made with it on the large scale, and have shown that its nutritive value corresponds to that of fresh milk.

